# Functional Biomarkers for Amyotrophic Lateral Sclerosis

**DOI:** 10.3389/fneur.2018.01141

**Published:** 2019-01-04

**Authors:** William Huynh, Thanuja Dharmadasa, Steve Vucic, Matthew C. Kiernan

**Affiliations:** ^1^Brain and Mind Centre, University of Sydney, Sydney, NSW, Australia; ^2^Prince of Wales Clinical School, University of New South Wales, Sydney, NSW, Australia; ^3^Western Clinical School, University of Sydney, Sydney, NSW, Australia; ^4^Institute of Clinical Neurosciences, Royal Prince Alfred Hospital, Sydney, NSW, Australia

**Keywords:** amyotrophic lateral sclerosis, motor neuron disease, neurophysiological biomarkers, transcranial magnetic stimulation, cortical excitability

## Abstract

The clinical diagnosis of amyotrophic lateral sclerosis (ALS) relies on determination of progressive dysfunction of both cortical as well as spinal and bulbar motor neurons. However, the variable mix of upper and lower motor neuron signs result in the clinical heterogeneity of patients with ALS, resulting frequently in delay of diagnosis as well as difficulty in monitoring disease progression and treatment outcomes particularly in a clinical trial setting. As such, the present review provides an overview of recently developed novel non-invasive electrophysiological techniques that may serve as biomarkers to assess UMN and LMN dysfunction in ALS patients.

## Introduction

Amyotrophic lateral sclerosis (ALS) is a rapidly progressive neurodegenerative disease that was first described in the 1869 by Jean-Martin Charcot (1–3) although earlier detailed clinicopathological descriptions of a case of ALS, was published by Radcliffe and Clarke (4). Charcot postulated the importance of the upper motor neuron in its pathogenesis (3) and its associated degeneration of motor cortical Betz cells that has become a well-recognized pathological feature (5, 6). The diagnosis of classical amyotrophic lateral sclerosis (ALS) relies on the clinical identification of progressive dysfunction in both the cortical (“upper”, UMN) and spinal (“lower”, LMN) motor neurons involving multiple body regions, much of which is encompassed within the El Escorial criteria (7, 8). The clinical heterogeneity of ALS is a result of the variable mix of UMN and LMN signs (9), hence contributing to delay in diagnosis and difficulty in monitoring disease progression as well as treatment outcomes particularly in a clinical trial setting (6). As such, there is a critical need to devise objective biomarkers of disease progression in ALS that may facilitate both improvement in diagnosis as well as to provide meaningful outcome measures to monitor treatment (10).

The present review will provide an overview of recently developed neurophysiological biomarkers, with emphases on novel non-invasive electrophysiological techniques used to assess UMN and LMN dysfunction in ALS patients.

### Biomarkers of UMN Dysfunction

An important component in the diagnosis of ALS relies on clinical features of UMN involvement in the presence of progressive LMN weakness (11), but often these signs of UMN impairment may be underappreciated in a limb that is concurrently affected by LMN loss especially in early stages of ALS (6, 12, 13). Upper motor neuron signs may initially be absent in approximately 7–10% of ALS patients (6, 14). As such, objective UMN biomarkers may be critical for the diagnosis of ALS, as potential mimicking disorders such as multifocal motor neuropathy, Kennedy's disease and adult-onset spinal muscular atrophy (SMA), may present as pure LMN syndromes (6, 15, 16). Autopsy reports have also demonstrated UMN degeneration in 50–75% of patients with clinically pure LMN syndromes (5, 17, 18).

### Transcranial Magnetic Stimulation

Since its original description more than 3 decades ago (19), Transcranial magnetic stimulation (TMS) has undergone significant evolution as a non-invasive technique for cortical stimulation, providing valuable insight into the functional integrity of brain pathways. Its main application has been in the investigation of complex neuronal networks of the primary motor cortex (M1), which is influenced by both inhibitory and excitatory mechanisms (20). Transcranial magnetic stimulation (TMS) biomarkers of cortical hyperexcitability appear to be useful biomakers of UMN dysfunction in ALS (21). In addition, TMS have provided insights into the underlying pathophysiological mechanisms in ALS, thereby allowing for the development of diagnostic and prognostic biomarkers in ALS (21).

**Figure 1:**
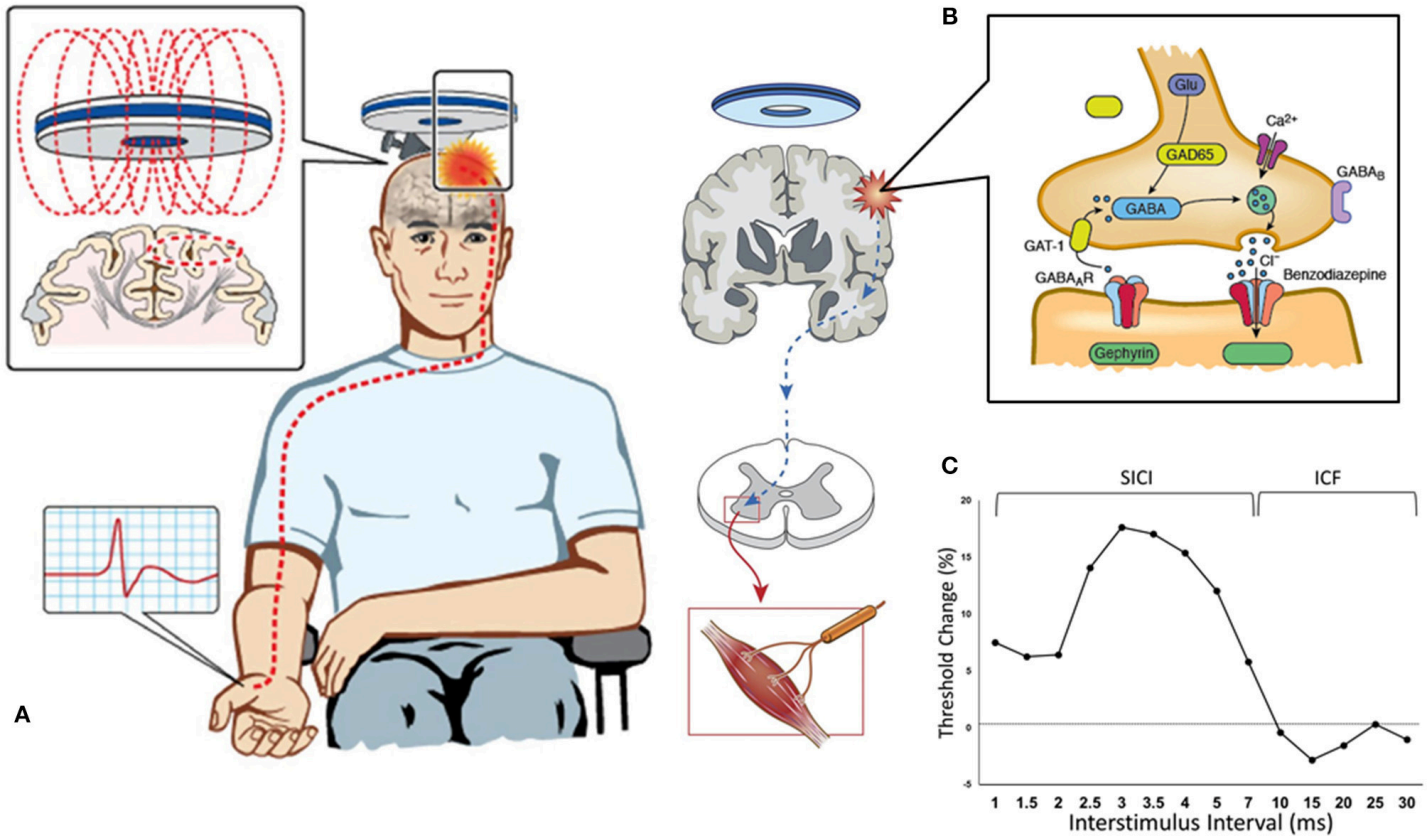
Paired-pulse threshold tracking transcranial magnetic stimulation (TMS). **(A)** TMS coil placed over the vertex activates the primary motor cortex and the response (motor evoked potential, MEP) is recorded from the contralateral abductor pollicis brevis muscle. **(B)** TMS parameters are mediated by a complex interplay between intraneural circuits and cortical output cells, with cortical interneurons mediating inhibition by activation of GABAergic synapses leading to influx of chloride anions (Cl^−^) and hyperpolarization of post-synaptic neurons. **(C)** Change in stimulus intensity required to achieve a target MEP of 0.2 mV (±20%) is used to quantify SICI (which is recorded with interstimulus intervals between 1–7 ms) and ICF (between 10–30 ms).

The TMS technique utilizes a transient magnetic field to induce an electric current in the cortex (22). This magnetic field is generated through a stimulating coil held over a subject's head, which painlessly and non-invasively penetrates the skull without attenuation (Figure 1). Depending on stimulation intensity and coil type, the electromagnetic force can stimulate neurons at a depth of 1.5–3.0 cm beneath the scalp (23). There have been several theoretical models postulated to explain the exact effect of this electromagnetic field on biological tissue, with studies in both animals and humans conferring that TMS generates a corticomotoneuronal volley composed of direct (D) and indirect (I) waves occurs at intervals of 1.5–2.5 ms (24). Direct waves are thought to represent the activation of corticospinal axons and are only recruited at high intensities or with the TMS coil positioned such that induces currents in a lateral-medial direction. Indirect waves seem to be activated at lower intensities and are mediated by a more complex interaction between cortical excitatory and inhibitory neurons (25). TMS delivered over the primary motor cortex (M1) is thought to activate pyramidal neurons (Betz cells) trans-synaptically via I-waves (26), but the exact neural circuitries evoked remain to be determined. These complex neural circuits are critically dependent on both excitatory and inhibitory interneuronal systems, facilitated by cellular receptor and neurotransmitter interactions (27). Excitation is primarily mediated by glutamate/NMDA receptor interaction, while inhibition is facilitated by γ-aminobutyric acid (GABA)/GABA_A/B_ receptor action (28).

Cortical hyperexcitability in ALS is heralded by reduced short-interval intracortical inhibition and CSP duration, in addition to increased intracortical facilitation and motor evoked potential amplitude (12, 29, 30). Furthermore, significant bilateral TMS abnormalities was observed in ALS patients at an early disease stage (31), consistent with previous studies that have reported functional abnormalities of the motor cortex as an early and specific feature of ALS, and preceding the onset of LMN dysfunction (6, 12, 29, 30, 32–34). More recent studies have demonstrated changes in TMS parameters indicative of cortical hyperexcitability, were more prominent over the dominant motor cortex and in particular, contralateral to the site of disease onset, suggesting a vulnerability of the dominant motor cortical neurons and supporting the importance of cortical processes in the pathophysiology of ALS as postulated first by Menon et al. (31).

### Single-Pulse TMS

The resting motor threshold (RMT) is a reflection of the ease with which corticomotoneurons are excited, hence the corticomotoneuronal membrane excitability, as well as the density of UMN projections onto motor neurons (35). Through the α-amino-3-hydroxy-5-methyl-4-isoxazoleproprionic acid (AMPA) receptors, RMT is influenced by the glutamatergic neurotransmitter system, such that excessive glutamate activity reduces RMT, and is susceptible to modulation by sodium channel blockers (28, 36). In ALS, the RMT is reduced early in the disease (indicative of cortical hyperexcitability) followed by progressive increase and eventual inexcitability with disease progression (32, 37–39). As motor threshold is modulated by glutamate activity (28), the reduced motor threshold supports the notion that cortical hyperexcitability being an early feature of ALS contributing to the ensuing lower motor neuron degeneration (21). The motor cortex is found to be inexcitable in approximately 20% of ALS patients and appears to be a late finding. In contrast, motor cortex inexcitability is a relatively frequent fidning in patients exhibiting the pure UMN phenotype termed primary lateral sclerosis [PLS] (40).

The central motor conduction time (CMCT) time is defined by the time interval between stimulation of the motor cortex and arrival of the corticospinal volleys at the spinal motor neurons, and is inferred from the motor evoked potential (MEP) onset latency (21). Prolongation of CMCT is an invariable finding in ALS being documented in 16–100% across different series (5, 21, 37, 41–44). In patients without clinically predominant UMN phenotypes, prolongation of CMCTs occurs in 50–71% of patients (41, 44). Although the mechanisms underlying CMCT prolongation are presently not fully elucidated, an increase in desynchronization of corticomotoneuronal volleys resulting from degeneration of the fastest conducting corticomotoneuronal fibers has been suggested (45, 46). Large discrepancies in sensitivity of this parameter reported by previous studies are likely attributable to technique-dependent variations associated with CMCT calculations.

The cortical silent period (CSP) refers to the interruption of voluntary electromyography activity in a target muscle after motor cortex stimulation (47), and the mechanisms that underly the CSP are complex but thought to be mediated primarily by the activation of inhibitory neurons acting via GABA-B receptors within the cortex (21, 48). The CSP duration has been consistently reduced in patients across all ALS phenotypes (21, 30, 32, 34, 43, 49–51). The decrease in CSP duration in ALS patients likely represent a combination of degeneration of inhibitory interneurons as well as GABAB-mediated receptor inhibition dysfunction (21).

### Paired-Pulse TMS

In the paired-pulse paradigm, a conditioning stimulus (CS) precedes and is utilized to modulate the effect of a second test stimulus (TS). By varying the time interval between the paired pulses (the interstimulus interval, ISI) a number of parameters can be determined, using either a constant stimulus method [in which the CS and TS are kept at a constant level and MEP amplitude is evaluated (52)] or the threshold-tracking (TT) TMS protocol (53). TT-TMS was developed to overcome the marked MEP amplitude variability seen when utilizing the earlier protocol and uses a fixed MEP response which is tracked by a varying TS (53, 54). By applying a subthreshold (set at 70% RMT) conditioning stimulus at predetermined time intervals prior to a suprathreshold test stimulus, the threshold-tracking TMS technique allows the short-interval intracortical inhibition (SICI) and intracortical facilitation (ICF) to be investigated (53, 55) (Figure 1).

**Figure 2:**
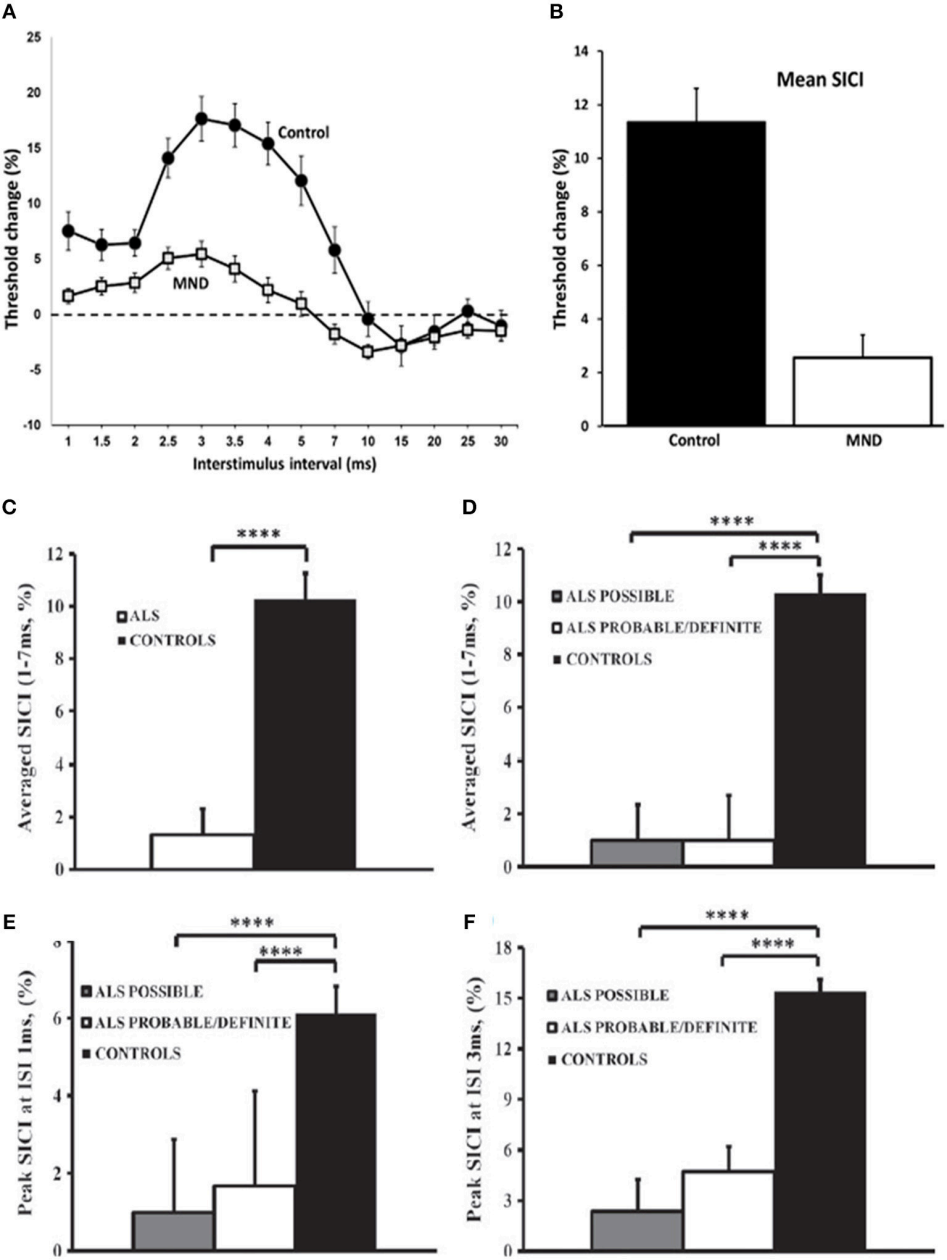
Cortical excitability in motor neuron disease (MND). Paired-pulse subthreshold conditioning transcranial magnetic stimulation demonstrating **(A)** reduction in short-interval intracortical inhibition (SICI, above dotted line) and intracortical facilitation (ICF, below dotted line) and **(B)** significant reductions in averaged SICI (between interstimulus intervals of 1–7 ms) in MND patients compared with controls **(C)** Averaged short-interval intracortical inhibition (SICI), between interstimulus interval (ISI) 1–7 ms, was significantly reduced in amyotrophic lateral sclerosis (ALS). **(D)** The reduction of averaged SICI was comparable in Awaji subgroups. Peak SICI at ISI **(E)** 1 ms, and **(F)** 3 ms was significantly reduced in Awaji subgroups. ****P < 0.0001. Reproduced with permission license no. 4457360494951 (1) and license no. 4457440155614 (12).

Reduction or absence of SICI, which is a biomarker of cortical interneuronal inhibitory GABAergic function, has been established as an early feature of ALS (Figures 2A,B), correlating with biomarkers of peripheral neurodegeneration and at times preceding the onset of LMN dysfunction in sporadic ALS cohorts [(31, 53), etc]. Although there were no significant differences in the degree of reduction observed between the sides of the motor cortices, there was a trend for more changes observed over the dominant motor cortex, particularly contralateral to the side of disease onset (31). The changes were also similar regardless of the severity of LMN dysfunction, or site of onset (bulbar or limb) (12, 21, 32, 56).

The reduction in SICI has been a widely reported feature present in both familial and sporadic forms of ALS with the alterations observed as an early feature (21, 30, 34, 57–62). Further to this, longitudinal assessments of asymptomatic SOD-1 mutation carriers have identified cortical hyperexcitability developing prior to the clinical onset of ALS, therefore suggesting that cortical hyperexcitability underlies the process of neurodegeneration in familial ALS (34).

The use of threshold-tracking TMS may be able to uncover UMN involvement in ALS phenotypes without clinically evident UMN signs such as the flail limb variant of ALS or progressive muscular atrophy (PMA). Moreover, this technique was able to reliably distinguish between ALS and other neurological mimic conditions including multifocal motor neuropathy, spinal muscular atrophy, Kennedy's disease, peripheral nerve hyperexcitability disorders, Hirayama disease, CIDP, lead neuropathy, hereditary spastic paraparesis, as well as hereditary motor neuropathy with pyramidal features (63–68).

SICI abnormalities using the threshold-tracking technique, appear to be the most robust diagnostic parameter that is indicative of UMN dysfunction in ALS patients (12, 29, 69). Using either an abnormal SICI or an inexcitable cortex, this TMS method demonstrated a sensitivity of approximately 73% and a specificity of 81% (69). Moreover, an absent SICI was associated with a 97% sensitivity (33). TMS abnormalities were observed in 77% of patients with ALS, with frequency of abnormalities that were similar across all Awaji diagnostic groups, using the established cut-off SICI of < 5.5% (63) resulting in 88% of Awaji-criteria possible patients being reclassified as Awaji-criteria probable or definite (12). More specifically, an abnormally reduced SICI was demonstrated in 56% of Awaji-criteria possible patients (12) (Figures 2C–F).

**Figure 3:**
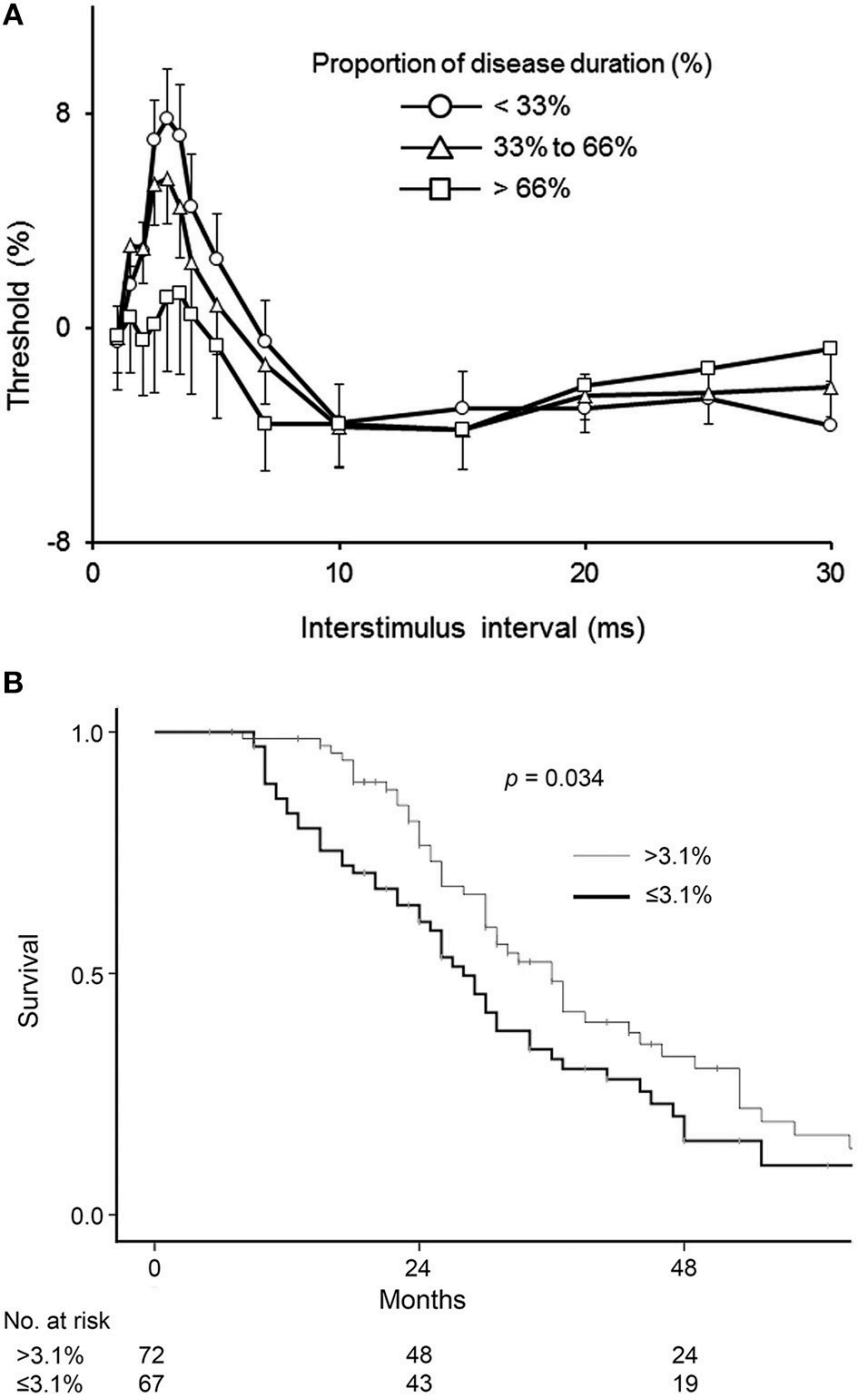
**(A)** Cortical excitability changes with disease progression. Patients were divided into three groups according to disease stage. The duration of the illness from onset to death was normalized between zero and one and expressed as a percentage (%), with data averaged by proportion of disease duration. Early stage (Circle) was defined as the proportion of disease duration < 33%, mid (Triangle) was 33–66%, and late (Square) was >66%. ALSFRS-R of patients in early stage was 42.3 ± 0.6, that in mid was 40.2 ± 0.7, and that in late was 34.8 ± 2.0. SICI at ISI 1–7 ms decreased with disease progression. Data are given as mean ± SE. Reproduced with permission license no. 4456860473754 (70). **(B)** Kaplan-Meier plots of survival probabilities according to averaged short-interval intracortical inhibition (SICI) values. Amyotrophic lateral sclerosis patients with a disease duration under 2 years were divided into 2 groups according to values in average SICI, interstimulus interval 1–7 ms. Patients with SICI ≤ 3.1% demonstrated reduced survival compared to patients with SICI >3.1% (*p* = 0.034). Estimated median survival was 28 months in patients with reduced SICI and 36 months in patients with higher SICI. Reproduced with permission license no. 4456870994973 (71).

More recent studies have also documented increasing cortical hyperexcitability with advancing disease indicating that intracortical inhibitory neurons become progressively dysfunctional in ALS (Figure 3A) (70). Reduced SICI was also reported to be an independent prognostic biomarker in ALS patients within the first 2 years of disease onset (71) (Figure 3B). Separately, SICI was shown to partially normalize with treatment by riluzole (72), an anti-glutamatergic agent exhibiting modest clinical effectiveness in ALS (73, 74). Paralleling the clinical efficacy Riluzole, the modulating effects last about 3 months (75), and may be related to overexpression of efflux pumps located at the blood brain barrier during the disease course (76). Regardless of the underlying mechanisms, studies of riluzole have suggested a utility of threshold-tracking TMS in assessing biological effectiveness of compounds at an early stage of drug development. Taken together, these results suggest that non-invasive *in vivo* monitoring of cortical function and particularly, SICI may also be an effective biomarker used to monitor the effects of novel therapeutics in a clinical trial setting.

### Biomarkers of LMN Dysfunction

Objective assessment of LMN dysfunction, utilizing neurophysiological techniques, appear to be more sensitive than clinical assessments (77, 78). Conventional neurophysiological techniques, such as nerve conduction studies which measure the compound muscle action potential (CMAP) amplitude, may be relatively insensitive in assessing LMN degeneration due to the process of reinnervation (79).

### Estimation of Motor Unit Numbers

**Figure 4:**
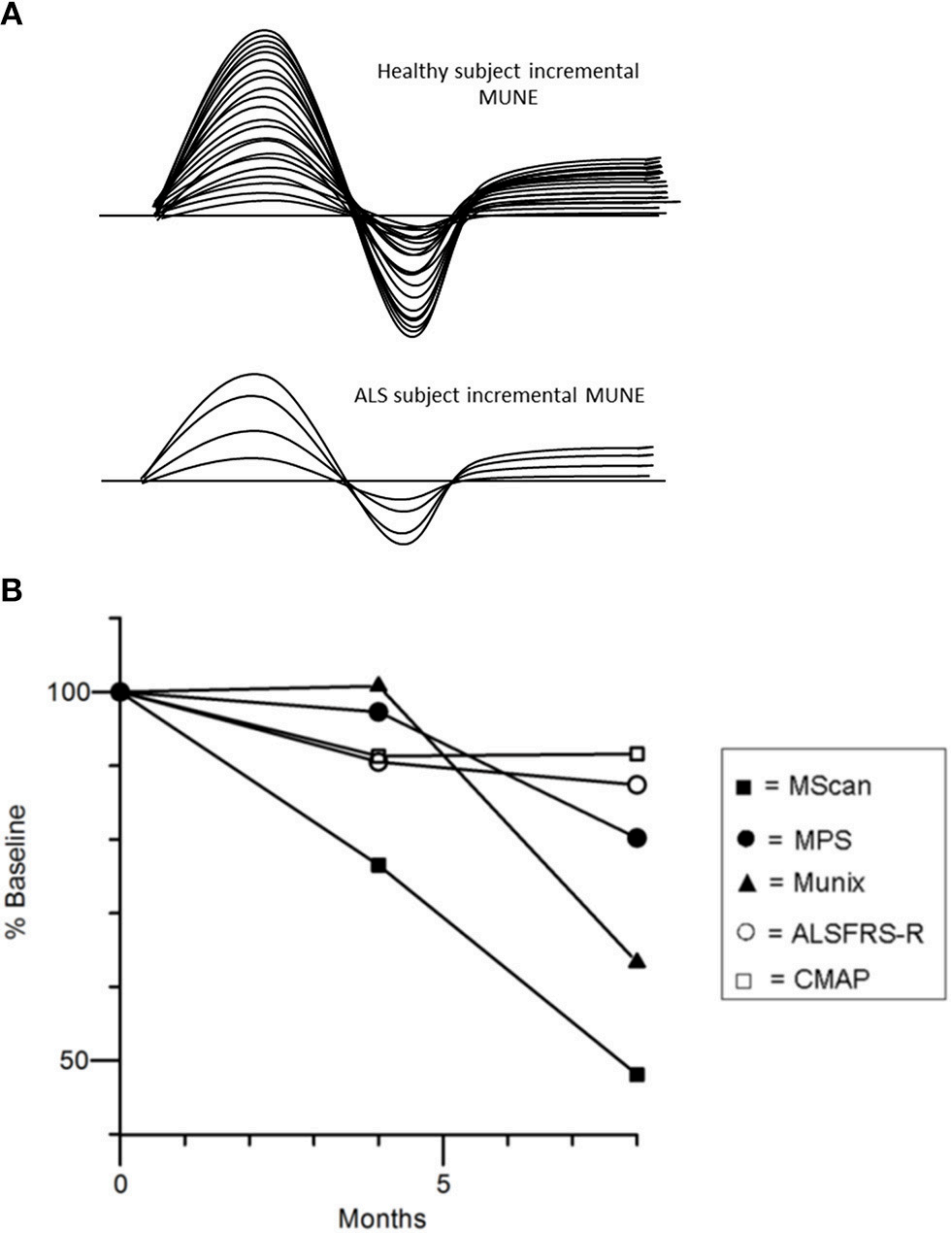
**(A)** Incremental MUNE in healthy and ALS subjects, demonstrating a large number of “steps” with increasing stimulus intensity consistent with a large number of functioning motor units within measured muscle, whilst there were only four steps in the ALS subject indicating only four functional motor units remaining in muscle. **(B)** Percentage changes in MUNE values (geometric means) and mean ALSFRS-R and CMAP amplitude at 4 and 8 months. Reproduced with permission license no. 4457481173441 (87).

As such, various methods to approximate the number of motor units innervating individual muscles, including motor unit number estimation (MUNE), and motor unit number index (MUNIX), may potentially represent valuable biomarkers of LMN degeneration. Since the development of the first MUNE technique in 1971 (80), there have been numerous other MUNE techniques introduced (81–85). The basic principle of MUNE techniques is the dividing of the maximal CMAP amplitude by the average surface-recorded motor unit potential (86). The original MUNE technique utilized incremental stimulation whereby the stimulus intensity at one point on the nerve was gradually increased from subthreshold until 10 increments in the motor response was recorded, but this technique relied on the assumption that the smallest recorded potential using the surface electrode over a target muscle following minimal stimulation represented a single motor unit potential (Figure 4A). Consequently, the variance in the result MUNE was considerable and resulted not uncommonly in artificially lower MUNE counts (86, 88).

The motor unit index (MUNIX) technique is a method designed to express the number of functioning motor units within a muscle as an index, instead of providing a direct measure of their absolute numbers. It is based on patients performing a voluntary contraction at various intensity levels and surface interference patterns being captured and decomposed to obtain a normalized motor unit size, which is then in turn divided into the maximal CMAP value to obtain the MUNIX (86, 89, 90).

Recent studies using different MUNE methods have demonstrated potential utility for assessing disease progression in ALS patients as reflected by a progressive linear decline in MUNE counts (87, 91–94). Interestingly, a recently developed MUNE technique, termed MScan, appeared to be the most sensitive MUNE method in detecting ALS disease progression (Figure 4B) (87). Additionally, MUNIX was able to detect disease progression in presymptomatic muscles in ALS (95, 96), and changes longitudinally in these muscle groups appeared more sensitive to those changes in the revised ALS Functional Rating Scale (ALSFRS-R) (93).

### Neurophysiological Index

The neurophysiological index (NI) is a potential electrophysiological biomarker in assessing lower motor neuron loss in ALS (97). Using a simple formula, The NI has the advantage of using routine CMAP amplitude, F-wave frequency, and distal motor latency of the ulnar-nerve innervated abductor digit minimi (ADM) muscle and is more sensitive than the CMAP amplitude alone in demonstrating longitudinal lower motor neuron loss in ALS. NI was able to detect motor neuron loss in muscles of the presymptomatic limb in ALS patients as well as successfully tracking disease progression, demonstrating continued loss of functional motor units during this presymptomatic period, when weakness, atrophy, or fasciculations were not detectable to both patients and evaluating clinicians (78). The validation of NI as a clinically meaningful parameter in disease progression of ALS patients was also demonstrated longitudinally in the symptomatic muscles of patients that correlated with their ALSFRS-R decline (97, 98). Additionally, NI was able to detect deterioration that occurred over a short period of 4 weeks in ALS patients, hence enabling the utility of this index in a clinical trial setting (77). NI has favorable reproducibility and low intraindividual variability but amongst its limitations, the index is only restricted to the ADM muscle (which is less affected compared to other intrinsic hand muscles such as the APB and FDI, in keeping with the split hand pattern of wasting and weakness) (99) and requires persistent F-waves (that can be frequently absent in ALS) (78).

### Split-Hand Index

The split-hand sign is documented as an early and specific clinical feature in patients with ALS that is not characteristic in other commonly encountered clinical mimics (99, 100). It refers to the preferential wasting and weakness of the thenar complex muscles (APB and FDI) with relative preservation of the hypothenar muscle, ADM (99), and appeared to have a cortical origin with the corticomotoneuronal input to the thenar complex in ALS patients preferentially affected (101, 102). This clinical observation provided an opportunity to develop a simple neurophysiological biomarker to aid the diagnosis of ALS using conventional nerve conduction studies. The split-hand index (SI) was derived by multiplying the CMAP amplitude of the APB muscle by the FDI CMAP amplitude and then dividing the product by the ADM CMAP amplitude. It was demonstrated that a reduction in the split-hand index was consistent across ALS phenotypes but appeared most pronounced in those with limb-onset, and that a cut-off value ≤ 5.2 reliably differentiated between ALS and other neurological disorders (103).

### Electrical Impedance Myography

Electrical impedance myography (EIM) is a novel non-invasive form of testing to provide quantitative information on neuromuscular disorders that may be useful and reliable in assessing longitudinally the severity of a disease process (104–107). EIM utilizes a small, high-frequency electrical current applied across two electrodes positioned over a muscle, and the resulting surface voltages are measured between a second pair of electrodes, from which the resistive and capacitive properties of the tissue are obtained (86, 105). The advantage is that this technique does not rely on inherent electrical activity of the tissue (which conventional neurophysiological techniques do), but rather on how the tissue impacts the applied current, rendering the technique sensitive to structural and compositional changes in muscle such as denervation, reinnervation, myofiber atrophy and fat replacement within the muscle that occur in ALS (104). EIM values have been shown to correlate with standard clinical approaches including handheld dynamometry and MUNE (106, 107), and may be able to provide more than a five-fold reduction in sample size requirements for ALS clinical therapeutic trials over standard outcome measures such as the ALS functional rating scale-revised (ALSFRS-R) (108). Although EIM can detect changes early in the disease course of ALS as well as in clinically unaffected muscle groups (105), a limitation of EIM is that identified changes may not be able to differentiate ALS from other neuromuscular conditions (109, 110).

## Conclusion

Amyotrophic lateral sclerosis remains a devastating neurodegenerative disorder with a poor prognosis, much of which is attributable to frequent delays in diagnosis, an incomplete understanding of the underlying pathophysiological mechanisms, and the current lack of effective disease-modifying treatment available. As such, there is a critical need to devise accurate and reliable biomarkers to address the above shortfalls in current ALS management. The current review has presented recent developments in novel neurophysiological biomarkers that are able to effectively interrogate upper and lower motor neuron dysfunction and characterize their change over time with disease progression, thereby exhibiting the potential to improve diagnosis, as well as facilitating in the prognosis and monitoring of the effects of future therapeutic agents in a clinical trial setting.

## Author Contributions

WH drafted manuscript and made all necessary edits and revisions as well as obtained required copyright permissions for figures. TD, SV, and MK revised and edited manuscript.

### Conflict of Interest Statement

The authors declare that the research was conducted in the absence of any commercial or financial relationships that could be construed as a potential conflict of interest.
